# Association between TB delay and TB treatment outcomes in HIV-TB co-infected patients: a study based on the multilevel propensity score method

**DOI:** 10.1186/s12879-024-09328-7

**Published:** 2024-04-30

**Authors:** Rujun Liao, Lin Hu, Jie Yu, Ying Chen, Miaoshuang Chen, Jingmin Yan, Xin Li, Xinyue Han, Chunnong Jike, Gang Yu, Ju Wang, Qiang Liao, Lan Xia, Xuefei Bai, Jinhong Shi, Tian Jiang, Liang Du, Tao Zhang

**Affiliations:** 1 Center of Infectious Diseases, Research Center of Clinical Epidemiology and Evidence-Based Medicine, Innovation Insititute for Integration of Medicine and Engineering, West China Hospital, Sichuan University, Chengdu, 610041 Sichuan People’s Republic of China; 2 Department of Epidemiology and Health Statistics, West China School of Public Health, West China Fourth Hospital, Sichuan University, Chengdu, 610041 Sichuan People’s Republic of China; 3 Sichuan Center for Disease Control and Prevention, Chengdu, 610041 Sichuan People’s Republic of China; 4 Liangshan Center for Disease Control and Prevention, Xichang, 615000 Sichuan People’s Republic of China; 5 Editorial department of Journal of Sichuan University (Medical Sciences), Sichuan University, Chengdu, CN People’s Republic of China

**Keywords:** Tuberculosis, HIV, Treatment, Delay, Propensity score, Multilevel model

## Abstract

**Background:**

HIV-tuberculosis (HIV-TB) co-infection is a significant public health concern worldwide. TB delay, consisting of patient delay, diagnostic delay, treatment delay, increases the risk of adverse anti-TB treatment (ATT) outcomes. Except for individual level variables, differences in regional levels have been shown to impact the ATT outcomes. However, few studies appropriately considered possible individual and regional level confounding variables. In this study, we aimed to assess the association of TB delay on treatment outcomes in HIV-TB co-infected patients in Liangshan Yi Autonomous Prefecture (Liangshan Prefecture) of China, using a causal inference framework while taking into account individual and regional level factors.

**Methods:**

We conducted a study to analyze data from 2068 patients with HIV-TB co-infection in Liangshan Prefecture from 2019 to 2022. To address potential confounding bias, we used a causal directed acyclic graph (DAG) to select appropriate confounding variables. Further, we controlled for these confounders through multilevel propensity score and inverse probability weighting (IPW).

**Results:**

The successful rate of ATT for patients with HIV-TB co-infection in Liangshan Prefecture was 91.2%. Total delay (*OR* = 1.411, 95% *CI*: 1.015, 1.962), diagnostic delay (*OR* = 1.778, 95% *CI*: 1.261, 2.508), treatment delay (*OR* = 1.749, 95% *CI*: 1.146, 2.668) and health system delay (*OR* = 1.480 95% *CI*: (1.035, 2.118) were identified as risk factors for successful ATT outcome. Sensitivity analysis demonstrated the robustness of these findings.

**Conclusions:**

HIV-TB co-infection prevention and control policy in Liangshan Prefecture should prioritize early treatment for diagnosed HIV-TB co-infected patients. It is urgent to improve the health system in Liangshan Prefecture to reduce delays in diagnosis and treatment.

**Supplementary Information:**

The online version contains supplementary material available at 10.1186/s12879-024-09328-7.

## Background

The COVID-19 pandemic has led to significant changes in the prevalence of tuberculosis (TB) worldwide [1, 2]. Notably, about 167,000 people died of HIV-associated TB in 2022. The percentage of notified TB patients who had a documented HIV test result in 2022 was 80%, up from 76% in 2021 [3], thus imposing a substantial public health burden [4–6]. China is classified as one of the 30 high-burden countries in terms of HIV-associated TB, with most of the cases coming from Liangshan Yi Autonomous Prefecture (Liangshan Prefecture) [7], which faces challenges due to its remote location, limited access to medical services and low residents health literacy [8, 9]. As a result, prioritizing prevention and control in Liangshan Prefecture is essential to achieve WHO’s global TB elimination goal by 2035 [10–12].

TB delay, consisting of patient delay, diagnostic delay, treatment delay, increases the risk of adverse anti-TB treatment (ATT) outcomes [13–17]. Also, HIV-positive patients experience greater delays in ATT compared to HIV-negative patients [18]. Therefore, reducing TB delay in HIV-TB co-infected patients is crucial. Additionally, infectious diseases are influenced by multiple factors, particularly tuberculosis, in which TB delay at various stages is impacted by a myriad of complex factors [19, 20]. Except for individual level variables, differences in economic level [14, 21] and healthcare coverage [5, 22] at regional levels have been shown to impact the ATT outcomes [23]. However, previous studies failed to adequately account for the individual and regional confounding variables that need to be controlled for when estimating the effects of different TB delays and ATT outcomes [24–26]. Directed acyclic graph (DAG) has been used to identify appropriate adjustment strategies [27], and propensity score methods as proposed by Rosenbaum and Rubin [28], specifically the use of inverse probability weight (IPW), have been suggested as approaches to address confounding and achieve unbiased exposure effect estimates. These methods were applied in a study, but this study also did not consider regional level variables [29]. Recently, multilevel propensity score was proposed to control for regional level confounding variables, indicating the necessity to be used in TB delay studies [30, 31].

Our study comprehensively analyzed the association between TB delay and TB treatment outcomes in HIV-TB co-infected patients in Liangshan Prefecture, China, using a causal inference approach. This is the first study to focus on HIV-TB co-infected patients, employing techniques like DAG, multilevel propensity score, and inverse probability weighting to address confounding variables at both the individual and regional levels. These methods improve the accuracy of estimating the association between TB delay and treatment outcomes.

## Methods

### Source of data and collection

Data on cases of HIV-TB co-infection in Liangshan Prefecture were collected from 17 counties (cities) from January 1, 2019, to December 31, 2022. The data was obtained from the special TB reporting system of 18 HIV-TB designated hospitals in the prefecture under license. The collected information included: (1) basic demographic details such as sex, ethnicity, age, education level, and marital status; (2) clinical information including the timing of ART initiation, timing of ATT initiation, TB symptoms, timing of initial TB diagnosis, treatment classification, sputum smear result, sputum culture result, CD_4_ cell count, and HIV viral load during follow-up; (3) socio-ecological factors at the county (city) level such as the medical personnel per 1,000 people and medical institutions per 1,000 people, as well as per capita medical consumption expenditure.

**Fig. 1:**
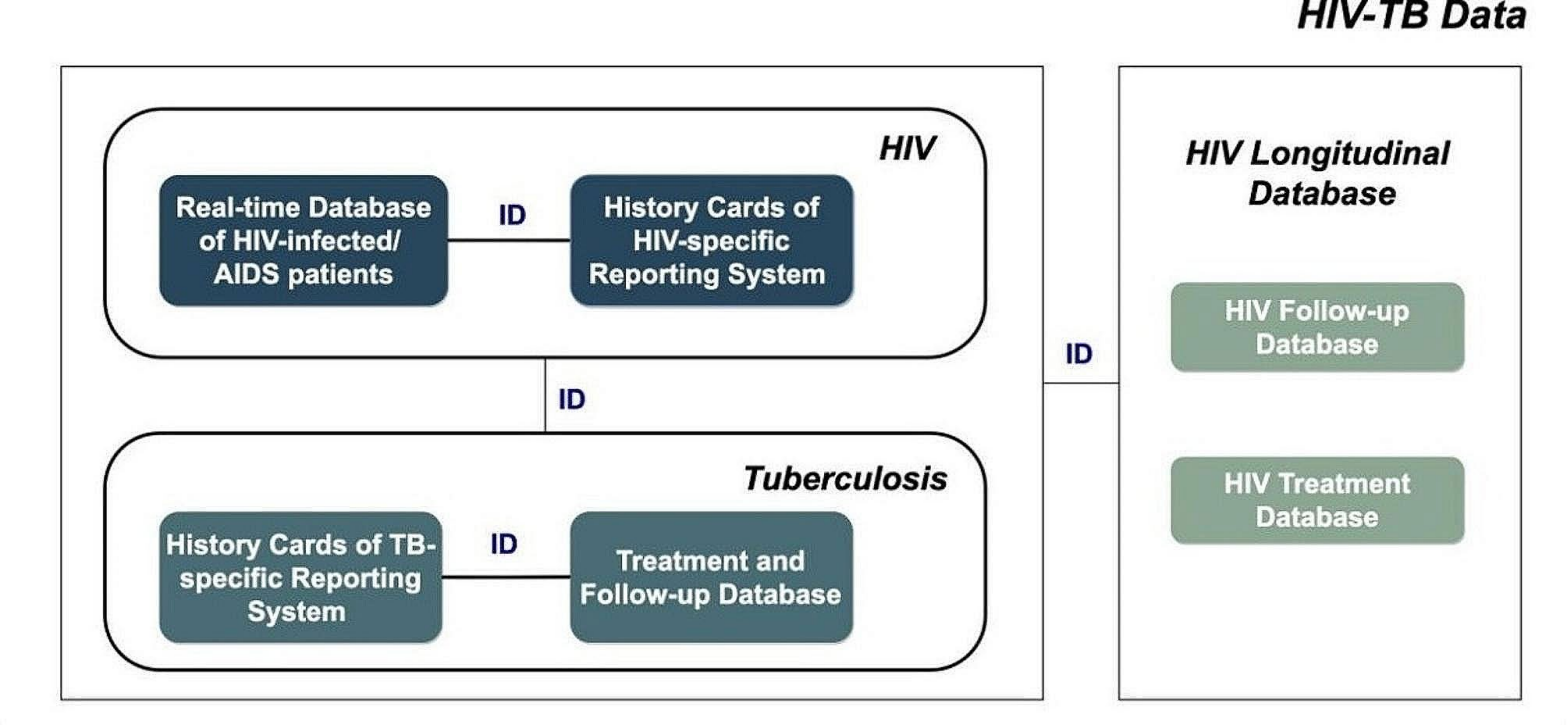
Data processing flow chart

All HIV-TB co-infection cases were included in the study except for those who did not belong to the Liangshan Prefecture area and those who were missing data on patient delay, diagnostic delay, treatment delay, treatment outcomes, and county (city) variables. After applying these exclusions, a total of 2068 co-infected patients were included in the analysis (Fig. 1). This study has been approved by the ethics committee of the Sichuan Center for Disease Control and Prevention (reference number: SCCDCIRB-2023-008).

### Definition of study variables

#### Diagnostic criteria for TB and AIDS

The diagnosis of TB in this study followed the “People’s Republic of China Health Industry Standard Tuberculosis Diagnosis WS 288–2017” [32], which aligned with the criteria established by the WHO [33]. Cases were classified as either clinical diagnosis or confirmed cases, with confirmation based on pathogenic and pathological results. Similarly, the diagnosis of AIDS was conducted according to the “China AIDS Diagnosis and Treatment Guidelines (2021 Edition)”, which also aligned with WHO criteria [34]. In this case, the diagnosis of HIV infection was based on the presence of HIV antibodies and etiological tests.

### Definitions related to TB delay

TB delay, as defined with reference to USAID [35], included TB patient delay, TB health system delay, and TB total delay. Health system delay could be subdivided into diagnostic delay and treatment delay. Patient delay was determined by calculating the time between the onset of TB symptoms and the first visit for these symptoms. Diagnostic delay was calculated as the time between the patient’s approach to the health system and TB diagnosis. Treatment delay was defined as the time between TB diagnosis and the initiation of ATT. Total delay encompassed the time between symptom onset and the initiation of ATT [35]. To establish cut-off values for TB delay, we consulted with clinicians and disease control specialists to identify values that were both clinically and statistically significant, and also referenced the literature [36, 37]. In this study, the cut-off values for TB delay defined as follows: 31 days as the cut-off value for patient delay (< 31 days; ≥31 days); 3 days as the cut-off value for diagnostic delay (< 3 days; ≥3 days); 1 day as the cut-off value for treatment delay (< 1 day; ≥1 day) ; 4 days as the cut-off value for health system delay (< 4 days; ≥4 days); and 34 days for total delay (< 34 days; ≥34 days).

### Definition of ATT outcome

According to the “Technical Specification for Prevention and Control of Tuberculosis in China (2020 Edition)” [38], which aligned with WHO criteria [33], ATT outcomes of TB patient were divided into two categories: successful ATT and unsuccessful ATT. Successful ATT encompassed both cure and completion of treatment. On the other hand, unsuccessful ATT encompassed other outcomes such as treatment failure, death, and missed visits.

### Definition of covariates

This study examined both individual-level and county-level confounding variables. At the individual level, we collected baseline TB-related clinical information, including symptoms, extrapulmonary involvement, comorbidities, and laboratory test results such as rifampin resistance, sputum culture, sputum smear, pathobiology assay test, CD_4_ count, HIV status, and viral load. At the county level, we considered covariates such as per capita medical consumption expenditure, medical personnel per 1,000 people, and medical institutions per 1,000 people. DAGs were plotted separately for patient delay, diagnostic delay, treatment delay, and total delay. DAGs [27] were used to identify the minimally sufficient set of confounders, which was determined using DAGitty [39] and the extended backdoor criterion. In total, 17 confounding covariates were included, and their definitions can be found in Additional file 1 (Table S1).

### Statistical analysis

Various techniques were employed to address missing data. In particular, the baseline CD_4_ count and HIV viral load were imputed using the closest follow-up data. For other covariates, a combination of multiple interpolation methods including logistic regression and polytomous regression imputation was utilized. When describing the baseline characteristics of the HIV-TB co-infection cases, categorical variables were summarized as numbers (percentages), and continuous variables as *M* (*P*_25_, *P*_75_).

To address confounding bias, we employed a doubly-robust approach, widely utilized for examining the relationships between health behaviors and outcomes [31]. Our analysis consisted of two stages for each TB delay. In the first stage, we utilized two-level logistic propensity score models and corresponding stabilized IPW to construct a pseudo population, ensuring covariate balance across exposure levels. In the second stage, a two-level logistic regression model was constructed to estimate the average treatment effect (ATE) of each TB delay, considering the covariates and exposures in the pseudo population [36]. Covariate balance was assessed by calculating the absolute standardized mean difference (ASMD). The effect of each TB delay was reported as *OR* with 95% *CI*. Assumptions of unconfoundedness, positivity, and stable unit treatment value assumption (SUTVA) were made to obtain unbiased estimations of the ATE [40].

We conducted a series of sensitivity analyses to evaluate the impact of selection bias and confounding on our findings. First, we explored different cut-off values for defining diagnostic delay, treatment delay and total delay. Second, we employed a Bayesian additive regression trees (BART) model with random effect terms for constructing the propensity score model, to validate the model construction robustness [30]. Third, we calculated the *E*-value to quantitatively assess the potential bias caused by unmeasured confounding. The *E*-value represented the minimum strength that an unmeasured confounder would need to be associated with both TB delay and treatment outcomes to explain away the findings of our study, while considering all measured covariates [41]. The results of the sensitivity analysis were presented in the additional file 1.

All statistical analyses were performed using R 4.3.2, and a significance level of 0.05 was used.

## Results

### Basic characteristics of the patients

A total of 2068 patients with HIV-TB co-infection were included in the analysis. The median age of the patients was 37(32, 43) years, with a majority being male (1560, 75.4%). Among these co-infected cases, 308 patients (14.9%) had extrapulmonary TB, while only 160 patients (7.7%) had previously received TB treatment. Approximately half of the patients (1025, 49.6%) had at least 1 comorbidity, and the median CD_4_ count was 304.00(181.75, 467.00) cells/mm^3^ (Table 1). The median patient delay was 34 days, the median health system delay was 1 days, while the median diagnostic delay and treatment delay were both less than 1 day. The median total delay from symptom onset to initiation of treatment was 39 days. Successful and unsuccessful rates of ATT in the HIV-TB co-infection patients for each group are presented in Table 1.

**Table 1:** Characteristics of the HIV-TB study population(%)

Variables	Overall	Successful	Unsuccessful
**Sociodemographics**			
**Sex**			
Male	1560	1411 (90.4)	149 (9.6)
Female	508	474 (93.3)	34 (6.7)
**Age**			
≤ 29	439	414 (94.3)	25 (5.7)
30–44	1302	1176 (90.3)	126 (9.7)
45–59	267	245 (91.8)	22 (8.2)
≥ 60	59	49 (83.1)	10 (16.9)
**Ethnicity**			
Yi	111	105 (94.6)	6 (5.4)
Others	1957	1780 (91.0)	177 (9.0)
**Marital status**			
Unmarried	521	488 (93.7)	33 (6.3)
Currently married	1207	1097 (90.9)	110 (9.1)
Divorced/Widowed	299	260 (87.0)	39 (13.0)
**Education level**			
Illiterate or barely literate	1081	999 (92.4)	82 (7.6)
Primary school	741	671 (90.6)	70 (9.4)
Junior high school and above	246	215 (87.4)	31 (12.6)
**Occupation**			
Farmer/herdsman/fisherman	1622	1471 (90.7)	151 (9.3)
Student/nursery child/scattered children	87	82 (94.3)	5 (5.7)
Housework and unemployment/retirement	141	128 (90.8)	13 (9.2)
Peasant/worker	55	51 (92.7)	4 (7.3)
**Clinical characteristics**			
**Rifampin resistance**			
No	2024	1870(92.4)	154(7.6)
Yes	44	15(34.1)	29(65.9)
**Patient category**			
New cases	1908	1758 (92.1)	150 (7.9)
Re treatment	160	127 (79.4)	33 (20.6)
**Comorbidities**			
No	1043	950 (91.1)	93 (8.9)
Yes	1025	935 (91.2)	90 (8.8)
**Clinical symptoms**			
Not obvious	1875	1707 (91.0)	168 (9.0)
Obvious	193	178 (92.2)	15 (7.8)
**Extrapulmonary TB**			
No	1760	1634 (92.8)	126 (7.2)
Yes	308	251 (81.5)	57 (18.5)
**Sputum smear**			
Negative	1515	1412 (93.2)	103 (6.8)
Positive	472	406 (86.0)	66 (14.0)
**Sputum culture**			
Negative	452	425 (94.0)	27 (6.0)
Positive	355	308 (86.8)	47 (13.2)
**Pathobiology assay test**			
Negative	1254	1181 (94.2)	73 (5.8)
Positive	772	673 (87.2)	99 (12.8)
**Treatment sequence**			
ART first	1706	1561 (91.5)	145 (8.5)
ATT first	297	277 (93.3)	20 (6.7)
**CD**_**4**_ **count, cells per mm** ^**3**^			
< 200	583	513 (88.0)	70 (12.0)
200–499	1014	944 (93.1)	70 (6.9)
500–999	388	361 (93.0)	27 (7.0)
≥ 1000	47	38 (80.9)	9 (19.1)
**HIV viral load, copies per mL**			
< 200	1081	1008 (93.2)	73 (6.8)
≥ 200	425	381 (89.6)	44 (10.4)
**Long treatment of HIV**			
No	1164	1069 (91.8)	95 (8.2)
Yes	839	769 (91.7)	70 (8.3)
**Patient delay**			
< 31 days	876	808 (92.2)	68 (7.8)
≥ 31 days	1192	1077 (90.4)	115 (9.6)
**Diagnostic delay**			
< 3 days	1567	1426 (91.0)	141 (9.0)
≥ 3 days	501	459 (91.6)	42 (8.4)
**Treatment delay**			
< 1 days	1473	1372 (93.1)	101 (6.9)
≥ 1 days	595	513 (86.2)	82 (13.8)
**Health system delay**			
< 4 days	1480	1356 (91.6)	124 (8.4)
≥ 4 days	588	529 (90.0)	59 (10.0)
**Total delay**			
< 34 days	860	794 (92.3)	66 (7.7)
≥ 34 days	1208	1091 (90.3)	117 (9.7)
**Total**	2068	1885 (91.2)	183 (8.8)

The overall successful rate of ATT for patients with HIV-TB co-infection was 91.2%. The unsuccessful rates for treatment varied depending on the type of delay. Among total delay, the unsuccessful rate was 7.7% in the non-delayed group and 9.7% in the delayed group. In terms of patient delay, the unsuccessful rate was 7.8% in the non-delayed group and 9.6% in the delayed group. For diagnostic delay, the unsuccessful rate was 9.0% in the non-delayed group and 8.4% in the delayed group. For treatment delay, the unsuccessful rate was 6.9% in the non-delayed group and 13.8% in the delayed group. Lastly, for health system delays, the unsuccessful rate was 8.4% in the non-delayed group and 10.0% in the delayed group.

### Treatment duration analysis

In addition, a total of 129 patients who had received treatment for less than 5 months were identified. Among these patients, the majority (76 cases) resulted in deaths, with 13 attributed to TB and 63 to causes unrelated to TB. Of the 53 surviving patients, 31 were referred to multidrug-resistant therapy and the remaining were a result of missed visits, occurrence of adverse events, or referrals to external institutions outside of Liangshan Prefecture. The treatment duration in our study ranged from 1 to 22 months, with a median duration of 6 months.

### Selection of confounding variables

To address confounding and establish a baseline precision, a set of covariables were chosen for inclusion in the propensity score based on the DAG (Additional file 1: Figures S1-S3). These covariables encompassed various factors such as age, sex, ethnicity, marital status, education level, occupation, re-treatment, comorbidities, clinical symptoms, extrapulmonary TB, sputum smear, sputum culture, pathobiology assay test, treatment sequence, CD_4_ count, HIV viral load, long treatment of HIV, place of residence, and regional level variables including per capita medical consumption expenditure, medical personnel per 1,000 people, and medical institutions per 1,000 people.

The balance test demonstrated that the two-level propensity score method successfully achieved a high level of covariate balance, indicated by an ASMD < 0.10 across all covariates (Additional file 1: Figure S4).

### Results of ATE estimation

In our analysis of the effect, we found that a total delay of ≥ 34 days was associated with an increased risk of unsuccessful ATT, with an *OR* of 1.411 (95% *CI*: 1.015, 1.962). This means that individuals with a total delay of ≥ 34 days were 1.411 times more likely to experience unsuccessful TB treatment compared to those with a total delay of < 34 days. Our results indicated that a diagnostic delay of ≥ 3 days was associated with a 1.778(1.261, 2.508) times higher likelihood of unsuccessful treatment compared to a diagnostic delay of < 3 days. Similarly, a treatment delay of ≥ 1 day was associated with a 1.749(1.146, 2.668) times higher likelihood of unsuccessful treatment compared to a treatment delay of less than 1 day. For health system delay, delay of ≥ 4 days was associated with a 1.480(1.035, 2.118) times higher likelihood of unsuccessful treatment compared to a diagnostic delay of < 4 days. While the effect of patient delay did not reach statistical significance, the estimated *OR* remained above 1, suggesting that patient delay still has a detrimental impact on the successful ATT. The detailed results are presented in Table 2.

**Table 2:** ATE estimation results

TB delay^a^	cut-off value	Estimate	Std. Error	z	OR
Total delay^b^	34	0.344	0.168	2.049	**1.411(1.015, 1.962)**
Patient delay ^c^	31	0.242	0.163	1.480	1.274(0.924,1.755)
Diagnostic delay^d^	3	0.576	0.176	3.281	**1.778(1.261,2.508)**
Treatment delay^e^	1	0.559	0.216	2.591	**1.749(1.146,2.668)**
Health system delay^e^	4	0.392	0.183	2.148	**1.480(1.035,2.118)**

^a^Total delay = Patient delay + Health system delay = Patient delay + (Diagnostic delay + Treatment delay).

^b^Total delay models were adjusted for treatment sequence, sputum smear, sputum culture, sex, per capita medical consumption expenditure, pathobiology assay test, marital status, long treatment of HIV, HIV viral load, extrapulmonary TB, ethnicity, education level, clinical symptoms, CD_4_ count and age.

^c^Patient delay models were adjusted for treatment sequence, sex, per capita medical consumption expenditure, patient category, occupation, marital status, long treatment of HIV, HIV viral load, ethnicity, education level, clinical symptoms, CD_4_ count and age.

^d^Diagnostic delay models were adjusted for treatment sequence, sputum smear, sputum culture, patient category, pathobiology assay test, medical personnel per 1,000 people, medical institutions per 1,000 people, long treatment of HIV, HIV viral load, clinical symptoms and CD_4_ count.

^e^Treatment delay and health system delay models were adjusted for rifampin resistance, treatment sequence, sputum smear, sputum culture, patient category, pathobiology assay test, medical personnel per 1,000 people, medical institutions per 1,000 people, long treatment of HIV, HIV viral load, extrapulmonary TB, clinical symptoms, CD_4_ count, BMI and age.

## Discussion

This study aimed to estimate the association between delays in TB diagnosis and treatment outcomes in patients co-infected with HIV and TB, using a causal inference perspective. The results demonstrated a 91.2% successful rate of ATT for HIV-TB co-infection patients in Liangshan Prefecture, which surpassed the WHO’s end TB strategy requirement [4]. Moreover, this successful rate was higher than that observed in TB-HIV co-infected patients in Hubei (89.52%) [42], Henan (80.19%) [43], and Shaanxi (88.44%) [44] provinces. This improved outcome may be attributed to the effective HIV and TB prevention and control efforts implemented in Liangshan Prefecture. The integrated “1 + M + N” network model for AIDS and TB prevention and control developed in this region has played a pivotal role in achieving such high treatment success rates for HIV-TB co-infection patients, surpassing the rates reported by 121 countries in the 2019 Global TB report [45]. These findings suggest that the management model implemented in Liangshan Prefecture, with its unique challenges of remote locations, poverty, and low literacy levels, could serve as a valuable reference for other resource-limited areas facing high prevalence of TB and HIV.

We observed that total delay in initiating TB treatment had a significant impact on treatment outcomes in patients with HIV-TB co-infection. A total delay of ≥ 34 days was found to be a risk factor for unsuccessful ATT, with an *OR* of 1.411 (95% *CI*: 1.015, 1.962), compared to a total delay of < 34 days. This finding was consistent with a prospective study in Ethiopia by Abyot Asres et al. [46], which reported that a total delay of > 30 days was associated with a 1.92 times higher incidence of unsuccessful ATT (95% *CI*: 1.30, 2.81) compared to a total delay of < 30 days. Further analysis of the components of total delay revealed that treatment delay and diagnostic delay were the major contributors to the overall delay effect. A treatment delay of ≥ 1 day was associated with a 1.749 times higher incidence of unsuccessful ATT (95% *CI*: 1.146, 2.668) compared to no treatment delay. Shashi Kant et al. [47] also reported that a delay in treatment initiation by more than 7 days was associated with unfavorable treatment outcomes, such as treatment default, failure, or death (*OR* = 1.87, 95% *CI*: 1.11–2.93). Our study further found that diagnostic delay was associated with ATT outcomes, with an *OR* of 1.778 (95% *CI*: 1.261–2.508), similar to a study in South Africa that found an association between diagnostic delays > 30 days and TB mortality [48]. In addition, our finding that health system delay of more than 4 days increased the incidence of ATT outcomes, which was consistent with previous studies showing that health system delays were associated with increased risks of death, pneumonia, and the use of mechanical ventilation in patients with TB [48, 49]. The association between total delay, treatment delay, diagnostic delay, and ATT outcomes may be attributed to factors such as longer treatment delay, higher clinical severity, or the development of ATT resistance [14, 50], leading to a higher incidence of unsuccessful ATT. However, our study did not find an association between patient delay and ATT outcomes, which was inconsistent with the results from Yi Xie et al. [51]. Nevertheless, our findings aligned with the results of Poppy Evenden et al. [52], who suggested that patient delay may have other adverse effects on ATT outcomes, such as deterioration of patient health status and prolonged treatment duration. Moreover, Graeme Meintjes et al. suggested that diagnostic delay might play a more significant role in TB delay compared to patient delay [48], and emphasized the importance of active case finding in resource-poor and remote settings [53, 54].

The cut-off value for TB delay in our study differed from some of previous studies [48]. In our study, referring to prior research and expert opinions [36, 37], we defined the diagnosis delay as 3 days. Meanwhile, the median diagnosis delay in China’s most recent study was 1(0,8) day [55], which was similar to the median diagnosis delay in this study. Considering regional variations and differences in the study population, this study determined the cut-off value based on the opinions of local experts. TB latency in China was already significantly shorter compared to other countries [36]. Additionally, in our research area, Liangshan Prefecture, extensive AIDS screening has been carried out since 2017, with TB, Hepatitis C, and syphilis being prioritized as prevention and control diseases since 2021. As a result, TB patients in Liangshan Prefecture experienced shorter delays in detection and early treatment compared to other regions. Additionally, unlike most previous studies that have focused on TB patients, this study targeted HIV-TB co-infected patients as the study population. Thus, our study considered the unique situation in Liangshan Prefecture when setting the cut-off value for TB delay. Sensitivity analysis was also conducted, which demonstrated that as the delay days (diagnosis or treatment delay) increased, the *OR* for TB delay in relation to ATT outcomes also increased. This indicated the robustness of our findings. By setting the cut-off value according to the actual situation in Liangshan Prefecture, our study provided valuable insights for improving TB prevention and control efforts in the future.

Hence, it is necessary to implement targeted interventions to address the diagnostic and treatment delays in Liangshan Prefecture. Previous studies by Li et al. [56] and Sreeramareddy et al. [36] have identified various factors contributing to these delays in high TB burden areas, including facility-related factors (such as lack of healthcare providers and high treatment costs), geographic barriers, and provider-related factors (e.g., lack of expertise, inadequate referral system, and misdiagnosis). These findings can inform policy recommendations for managing HIV-TB co-infections in Liangshan Prefecture. Key strategies should involve increasing the number of trained healthcare professionals through incentivization and recruitment, providing regular and ongoing professional training for existing staff, establishing healthcare facilities in remote areas, and implementing regular HIV and TB screening by healthcare workers to facilitate early detection and minimize diagnostic delays.

This study has several strengths. First, Liangshan Prefecture is an ideal location for studying the treatment of HIV-TB co-infected patients due to its high incidence of infectious diseases, including AIDS and tuberculosis. Second, the use of a causal DAG helped identify confounding factors. Additionally, the use of multilevel propensity score and inverse probability weighting methods allowed for control of measured individual and regional-level confounding variables, while also accounting for unmeasured regional-level confounding, ultimately ensuring the reliability of the study. Finally, the study assessed the robustness of the results through sensitivity analyses, including cut-off value selection, model construction, and unmeasured confounding. Despite these strengths, there are a few limitations that should be noted. First, the timing of symptom onset was self-reported by patients during consultation, which introduces the possibility of recall bias. Secondly, the limited availability of previous studies on effect analysis of TB delay and ATT outcomes in HIV-TB co-infected patients makes it challenging to compare and confirm the findings, although it also highlights the novelty of the reported results. Lastly, while the results of this study provide valuable insights for improving clinical management, additional research is needed to explore the mechanisms through which delayed effects may impact patient treatment outcomes.

## Conclusions

The management of HIV-TB co-infection in Liangshan Prefecture can serve as a valuable model for other resource-limited areas with high prevalence of TB and HIV. This study underscores the significance of addressing diagnostic and treatment delays as crucial areas for intervention in TB and HIV care. Therefore, it is imperative to prioritize health system improvement in the Liangshan Yi area in order to mitigate diagnostic and treatment delays and improve the overall management of HIV-TB co-infection.

### Electronic supplementary material

Below is the link to the electronic supplementary material.

Supplementary Material 1

## Data Availability

The data that support the findings of this study are available from Liangshan Prefecture Center for Disease Control and Prevention but restrictions apply to the availability of these data, which were used under license for the current study, and so are not publicly available. Data are however available from the corresponding authors upon reasonable request and with permission of Liangshan Prefecture Center for Disease Control and Prevention.

## Declarations

### Supplementary Information

Additional file 1. This Additional file presents some results of this study. ^(2218 K, docx)^

## Abbreviations

Liangshan Prefecture: Liangshan Yi Autonomous Prefecture
OR: Odds ratio
95% CI: 95% confidence interval
TB: Tuberculosis
AIDS: Acquired immunodeficiency syndrome
HIV: Human immunodeficiency virus
HIV-TB: HIV-tuberculosis
MDR/RR-TB: Multidrug-resistant/rifampicin-resistant TB
WHO: World Health Organization
ATT: Anti-TB treatment
ART: Antiretroviral therapy
DAG: Directed acyclic graph
ATE: Average treatment effect
BART: Bayesian additive regression trees
