# Supplementary material for: Association between TB delay and TB treatment outcomes in HIV-TB co-infected patients: a study based on the multilevel propensity score method
Source: BMC Infect Dis. 2024 Apr 30;24:457. doi: 10.1186/s12879-024-09328-7 (PMC11061920; doi:10.1186/s12879-024-09328-7)
Supplement: Supplementary file 1 — Supplementary Material 1 [file 12879_2024_9328_MOESM1_ESM.docx]

Table S1. Variable Assignment Table.

|  | **Variables** | **Value** |
| --- | --- | --- |
| SOCIODEMOGRAPHICS | Sex | Male =1, Female =2 |
|  | Age | ≤29=1, 30-44=2,  45-59=3, ≥60=4 |
|  | Marital Status | Unmarried =0,  Currently married =1,  Divorced/ Widowed =2 |
|  | Ethnicity | Yi =1, Others =2 |
|  | Education level | Illiterate or barely literate =1, primary school =2,  Junior high school and above =3 |
|  | Occupation | Farmer/ herdsman/ fisherman =1, Student/ nursery child/ scattered children =2, Housework and unemployment/ retirement =3, Peasant/ worker =4 |
| CLINICAL CHARACTERISTICS | Rifampin resistance | No=0, Yes=1 |
|  | Patient category | New cases =1, Re treatment =2 |
|  | Comorbidities | No=0, Yes=1 |
|  | Clinical symptoms | Not obvious =0, obvious =1 |
|  | Extrapulmonary TB | No=0, Yes=1 |
|  | Treatment sequence | ART first=0, ATT first=1 |
|  | CD_4_ count, cells per mm^3^ | <200=1, 200-499=2,  500-999=3, ≥1000=4 |
|  | Sputum smear | negative=0, positive=1 |
|  | Sputum culture | negative=0, positive=1 |
|  | Pathobiology assay test | negative=0, positive=1 |
|  | HIV viral load, copies per mL | <200=0, ≥200=1 |
|  | Long treatment of HIV | Yes=0, No=1 |

**Directed Acyclic Graph (DAG) Used for Variable Selection**

To ensure accurate adjustment without under or overadjustment, we conducted a systematic literature review to identify potential confounders for our study. Following the 'Evidence Synthesis for Constructing Directed Acyclic Graphs' method, we constructed a directed acyclic graph (DAG) to represent the relationships between our variables. Using an extended version of the backdoor criterion, we determined the minimally sufficient set of confounders based on the DAG. To visualize and interpret the DAG, we utilized the software program DAGitty, which can be found at http://www.dagitty.net/dags.html.


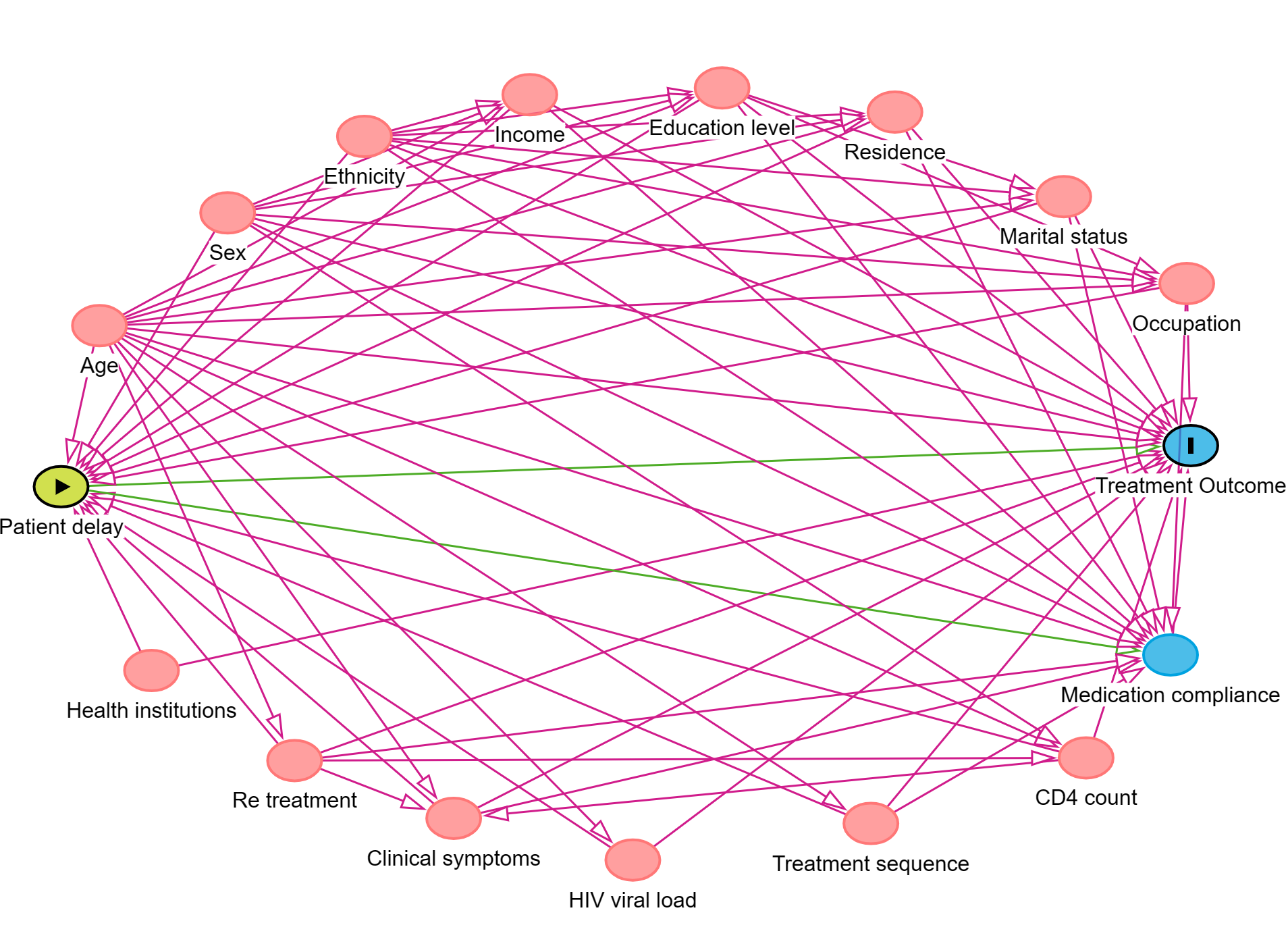


Figure S1. Patient delay DAG. All circles denote potential covariates and/or confounders, with blue ones also being potential mediators; all directed lines denote potential confounding effects, with green ones also denoting potential mediation effects.


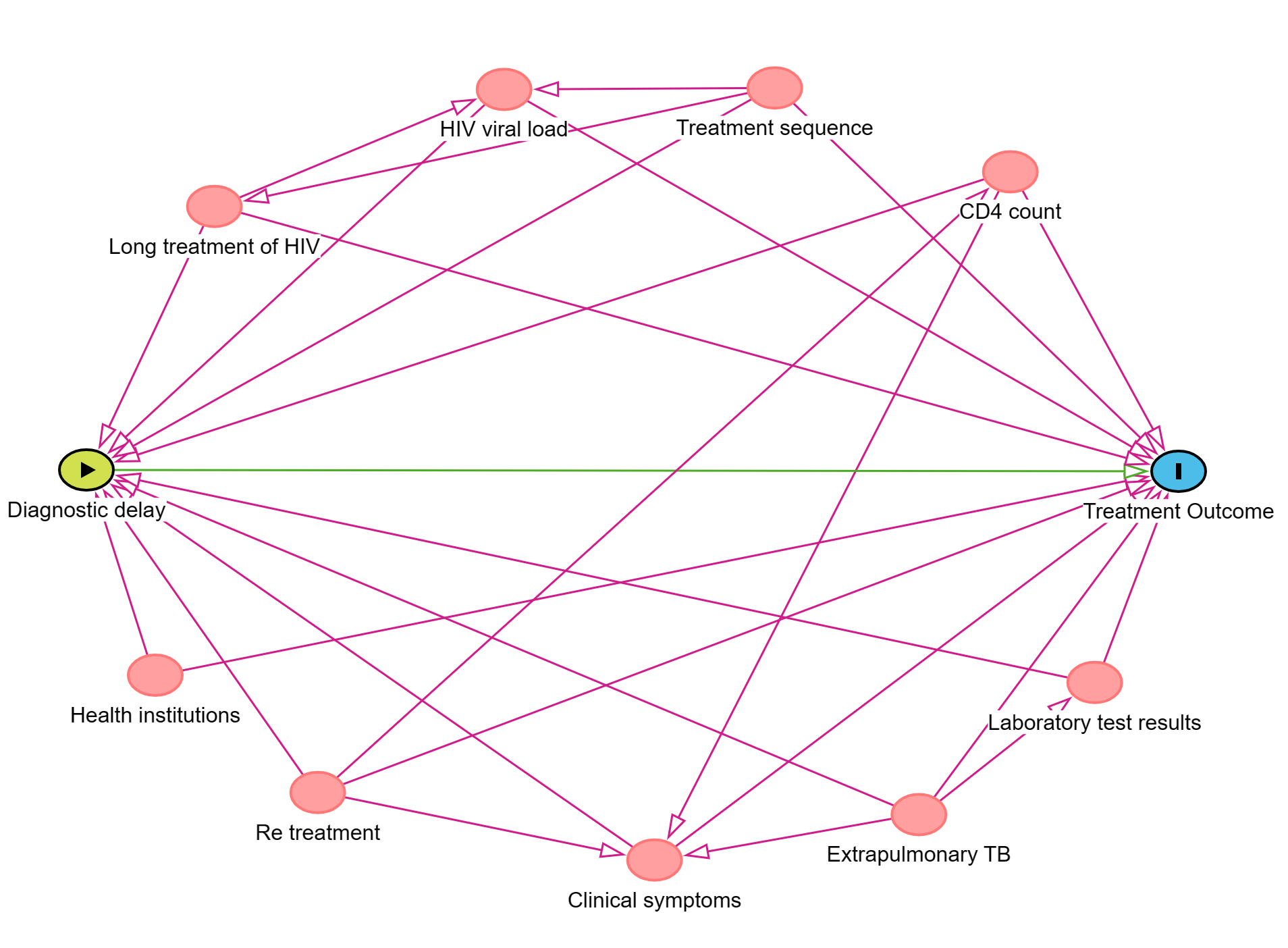


Figure S2: Diagnostic Delay DAG. All circles denote potential covariates and/or confounders, with all directed lines denote potential confounding effects.


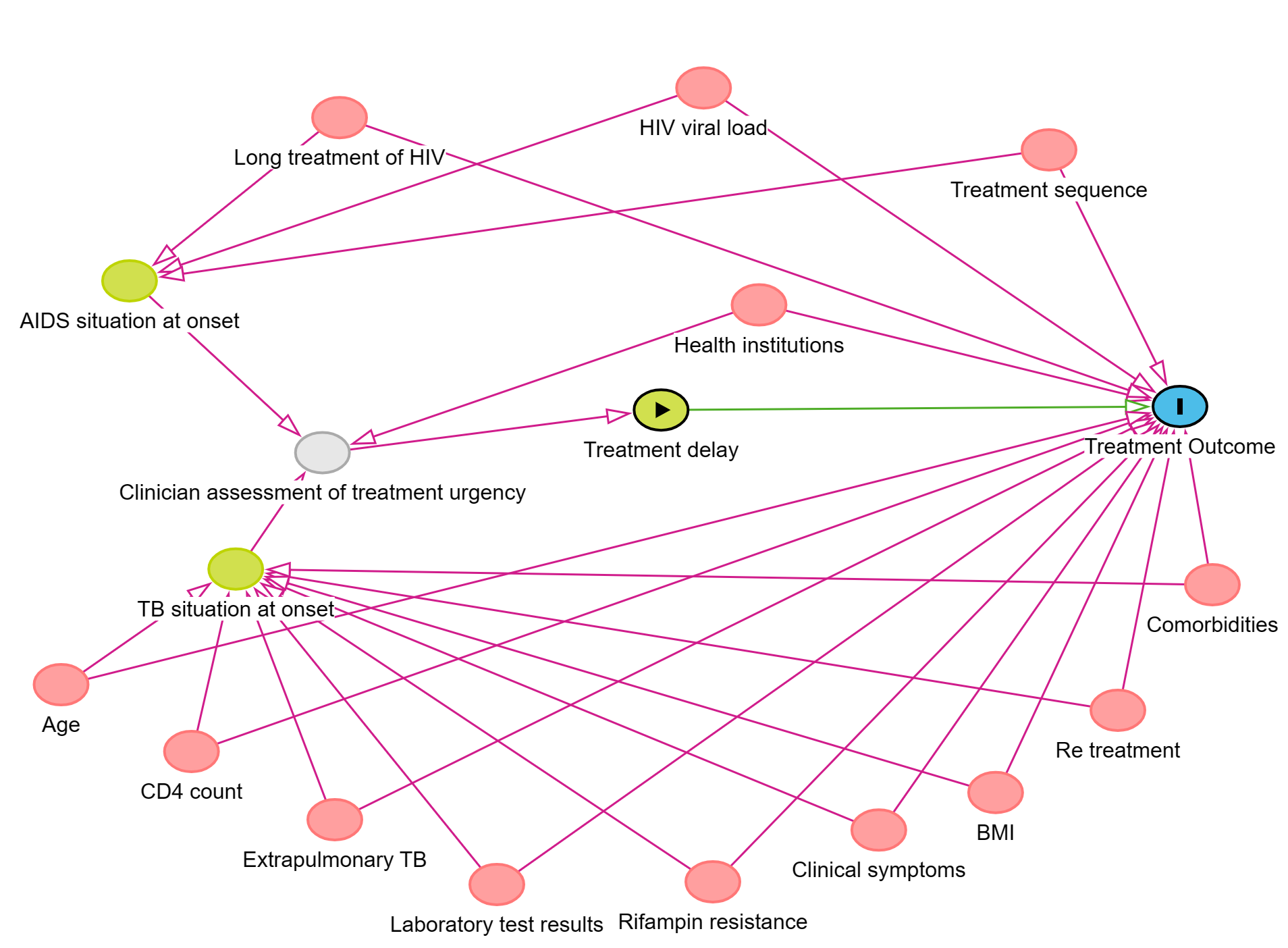


Figure S3: Treatment Delay DAG. All circles denote potential covariates and/or confounders, with all directed lines denote potential confounding effects.


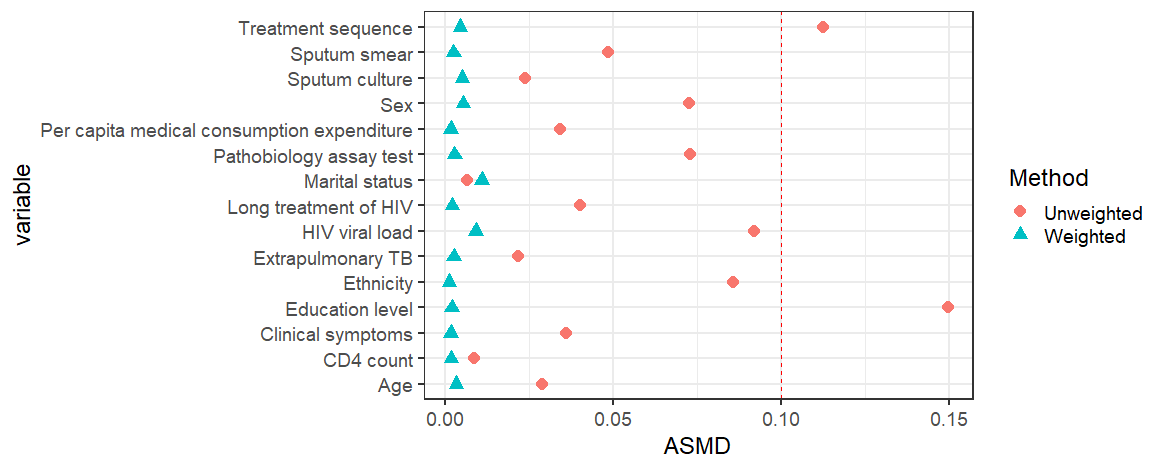


**a)**


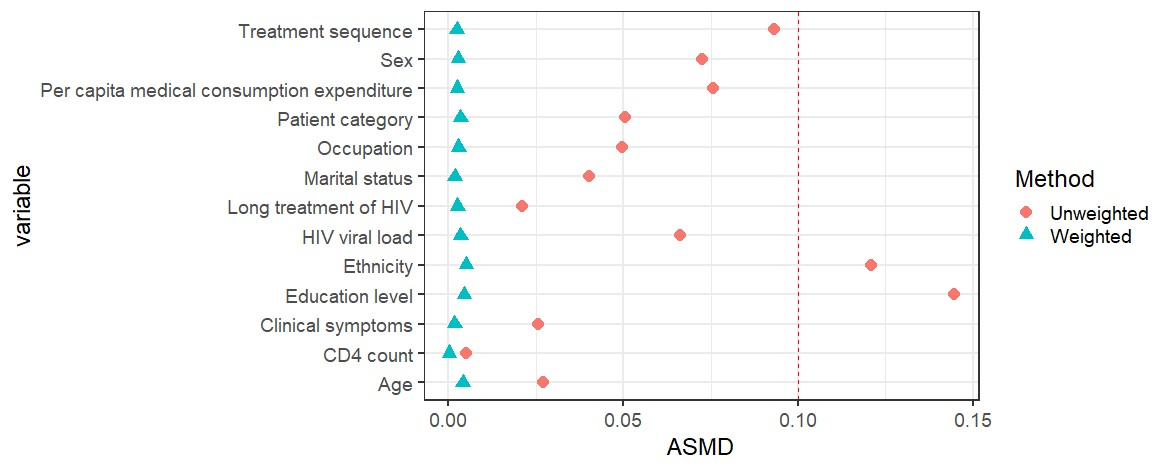

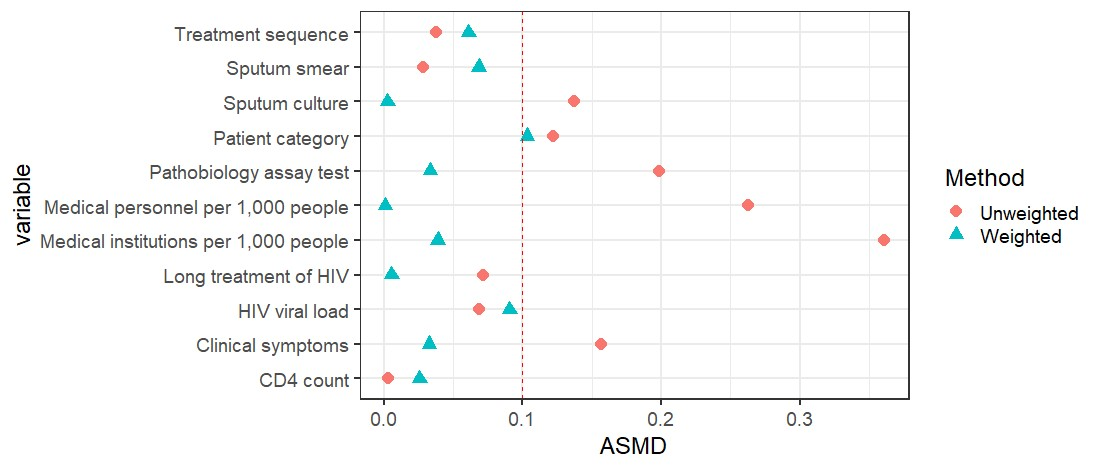


**b)**

**c)**


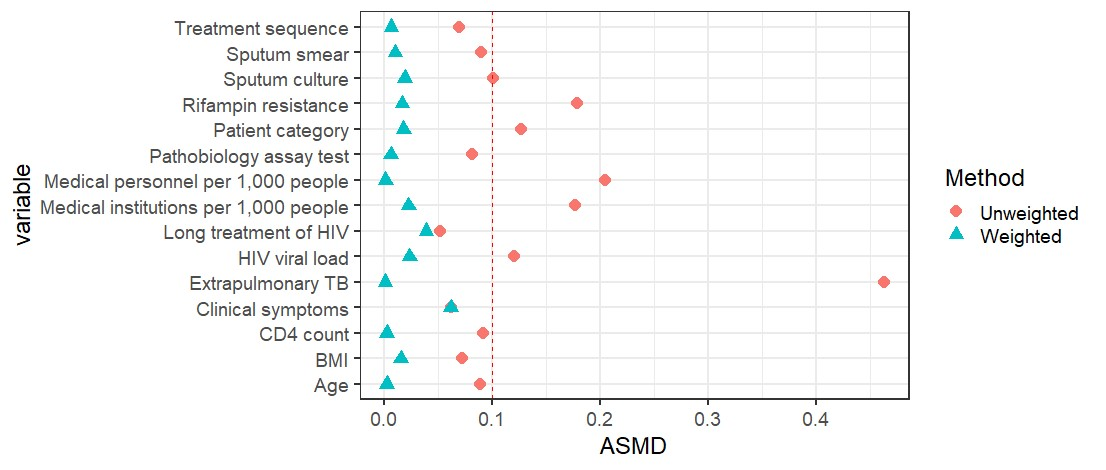


**d)**


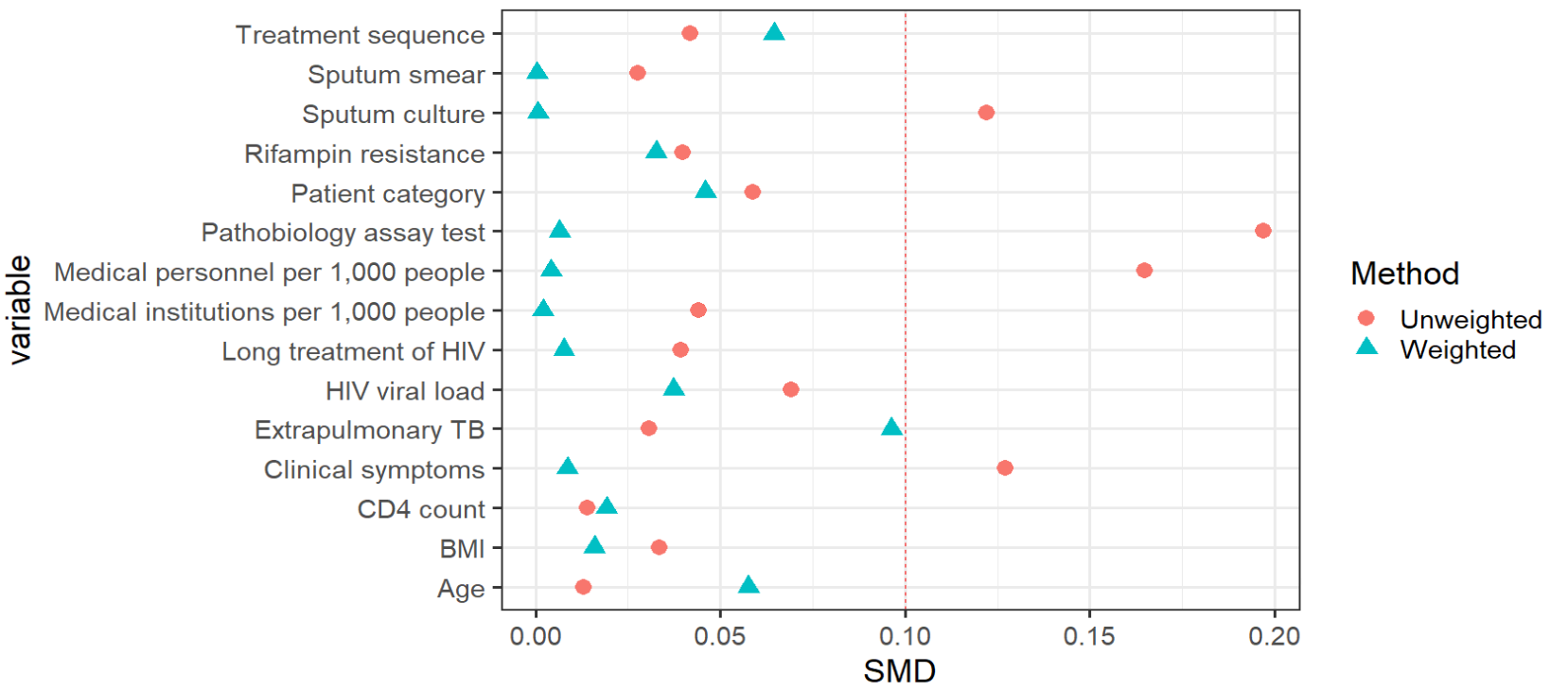


**e)**

**Figure S4. Balance assessment of Confounding covariates with delay effect as the exposure.** a) Total delay: marginal weights achieved confounding variable balance with an SMD of 0.0038. b) Patient delay: marginal weights achieved confounding variable balance with an SMD of 0.0029. c) Diagnostic delay: marginal weights achieved confounding variable balance with an SMD of 0.0420. d) Treatment delay: the marginal weights achieved confounding variable balance with an SMD of 0.0163. e) Health system delays: the marginal weights achieved confounding variable balance with an SMD of 0.0267.

**Sensitivity Analysis**

In this study, we conducted an analysis using alternative cut-off values for defining diagnostic delay and treatment delay, as presented in Table S2. Subsequently, a BART random-effects model was constructed for inverse probability weighting. The results of this analysis aligned with the earlier findings and emphasized the robustness of the study results. Additionally, the *E*-values were calculated for the ATE (average treatment effect) across various TB delays. These analyses revealed that only the odds ratios of unmeasured confounders surpassed the corresponding *E*-values, potentially influencing the effects of total delay, diagnostic delay, treatment delay, and health system delay identified in our study (refer to Table S3). Nevertheless, given the sufficient adjustment for confounding variables in our study, the likelihood of distorting the results due to unmeasured confounding factors appears to be relatively low.

Table S2. ATE estimation results for other cutoff values.

|  | cut-off value | Estimate | Std. Error | *z* | *OR* |
| --- | --- | --- | --- | --- | --- |
| Diagnostic delay | 2 | 0.315 | 0.168 | 1.880 | 1.370(0.987,1.903) |
|  | 1 | 0.137 | 0.159 | 0.862 | 1.147(0.840,1.566) |
| Treatment delay | 2 | 0.588 | 0.230 | 2.553 | 1.801(1.146,2.828) |
| Total delay | 35 | 0.320 | 0.166 | 1.924 | 1.378(0.994,1.909) |

Table S3. *OR* and *E*-values for different delays.

|  | *OR* | *E*-value |
| --- | --- | --- |
| Total delay | 1.411(1.015, 1.962) | 2.17(1.14) |
| Patient delay | 1.274(0.924,1.755) | 1.86(1.00) |
| Diagnostic delay | 1.778(1.261,2.508) | 2.95(1.83) |
| Treatment delay | 1.749(1.146,2.668) | 2.89(1.56) |
| Health system delay | 1.480(1.035,2.118) | 2.32(1.23) |

## References

1. WS 288-2017 Tuberculosis classification. <http://www.nhc.gov.cn/wjw/s9491/201712/a452586fd21d4018b0ebc00b89c06254.shtml>.

2. World Health Organization. Definitions and reporting framework for tuberculosis–2013 revision: updated December 2014 and January 2020. World Health Organization, 2013. <https://www.who.int/publications/i/item/9789241505345>

3. Chinese Medical Association. China AIDS Diagnosis and Treatment Guidelines (2021 Edition). <https://rs.yiigle.com/CN115673202105/1345215.htm>.

4. World Health Organization. WHO case definitions of HIV for surveillance and revised clinical staging and immunological classification of HIV-related disease in adults and children. World Health Organization, 2007.

5. General Office of the National Health Commission of the People 's Republic of China. Circular of the General Office of the National Health Commission on Printing and Issuing the Technical Specification for Prevention and Control of Tuberculosis in China (2020 Edition). National Health Office disease control letter [2020] No. 279.2020-04-02.

6. World Health Organization. Definitions and reporting framework for tuberculosis–2013 revision: updated December 2014 and January 2020. World Health Organization, 2013. <https://www.who.int/publications/i/item/9789241505345>
